# ADHD, Lifestyles and Comorbidities: A Call for an Holistic Perspective – from Medical to Societal Intervening Factors

**DOI:** 10.3389/fpsyg.2017.00454

**Published:** 2017-04-06

**Authors:** Simon Weissenberger, Radek Ptacek, Martina Klicperova-Baker, Andreja Erman, Katerina Schonova, Jiri Raboch, Michal Goetz

**Affiliations:** ^1^First Medical Faculty, Charles UniversityPrague, Czechia; ^2^Institute of Psychology, Czech Academy of SciencesPrague, Czechia; ^3^Faculty of Theology, University of LjubljanaLjubljana, Slovenia; ^4^Department of Child Psychiatry, Second Faculty of Medicine, Motol University Hospital, Charles UniversityPrague, Czechia

**Keywords:** ADHD, childhood, obesity, substance abuse, pollution, smoking, alcohol, comorbidities

## Abstract

The review examines Attention Deficit Hyperactivity Disorder (ADHD in its Child and Adult form) and its various presentations (Hyperactive Impulsive, Inattentive, and Combined) with a particular focus on environmental (incl. social factors), lifestyles and comorbidities. It is argued that ADHD is best understood in a holistic and interactive context and a vast empirical literature is presented to illustrate the point: Environmental factors include stress in general as well as exposure to toxins (phthalates, bisphenol A). Social factors are illustrated by effects of social deprivation and seduction to unhealthy lifestyles. Maternal lifestyle during pregnancy is pointed out (particularly her exposure to nicotine, alcohol, caffeine, and drugs, even seemingly benign medications like acetaminophen), which all tend to be related to ADHD. Family environment is discussed with respect to protective effect of (mainly authoritative and autocratic) parenting styles. Societal factors include mainly economic and political issues: income inequality and poverty (low SES is an ADHD risk factor) and a growing moral dilemma between a humanistic effort to globally spread the knowledge of ADHD and the medicalization and commercialization of the disorder. The second part of the review is devoted to ADHD related lifestyles and resulting comorbidities (e.g., food addiction and obesity, substance abuse, electronic media dependencies and conduct and personality disorders). Although ADHD is a neurodevelopmental disorder, its assessment and treatment are also linked to environmental, behavioral and social factors and their interactions.

## ADHD and its Incidence

Attention Deficit Hyperactivity Disorder (ADHD) is a lifelong neurodevelopmental disorder marked by its observable behavioral manifestations. The continuum of the symptoms is not always clear and ADHD may or may not continue into adulthood. The disorder presents itself as ‘childhood ADHD’ and ‘adult ADHD,’ with childhood ADHD being more common (Polanczyk et al., 2007). The disorder carries into adulthood approximately 50% of the time (Moreno-Alcázar et al., 2016) but the processes of remission still remain unclear. As of 2013, with the introduction of DSM-5, it is no longer classified as a childhood disorder but as a chronic lifelong disorder.

The marked symptoms of ADHD include general inattention, hyperactivity, impulsivity, and difficulty in self-control. These are seen in the various presentations of the disorder and they are divided in what was once called subtypes of the disorder but are currently referred to as presentations; they are ‘Hyperactive Impulsive’ (ADHD-HI) ‘Inattentive’ (ADHD-I), and ‘Combined’ (ADHD-C) (American Psychiatric Association, 2000). Importantly, the categories are not necessarily permanent as the disorder progresses: for example, a child with combined hyperactive and inattentive ADHD may later become less hyperactive and more inattentive with age if the disorder persists (Larsson et al., 2011).

There is a general agreement that the disorder disproportionately affects males compared to females but the gender proportion greatly differs among different studies; e.g., Polanczyk et al. (2007) assess the rate at 2.4–1.

Data from 2007 relevant to ADHD worldwide found childhood ADHD to be around 5.3% with lower rates in lower income nations and 3.4% incidence for Adult ADHD. The authors emphasized the importance of studying Adult ADHD to a greater extent, as this was before the DSM-5 classification (Fayyad et al., 2007). The progression of the disorder is often non-linear with remission of symptoms from childhood to adolescence occurring at times. From early childhood ADHD is associated with certain comorbidities such as Oppositional Defiant Disorder (ODD) and executive dysfunction, in a longitudinal study it was found that high oppositional traits and more severe clinical presentations along with family factors such as having a father with anxiety and internalizing disorders was a strong predictor for ADHD persisting into adolescence. Interestingly these factors were a better predictors than childhood adversity or cognitive functioning (McAuley et al., 2014).

Attention Deficit Hyperactivity Disorder is considered the most genetically anchored mental disorder (Ptacek et al., 2011b) with probabilities of up to 77% for both childhood and adult ADHD and around 30–40% in case of Adult ADHD only. In the case of Adult ADHD it is considered one of the most devastating disorders which in the long term may affect all areas of one’s life (Franke et al., 2012). Along with new neurological findings differentiating ADHD individuals from normally developing children and adolescents, the disorder is mostly recognized and diagnosed by its distinctive behavioral appearance. That leads to a wide variety of distinct documented behavioral traits or even lifestyles which are often attributable to a variety of comorbidities.

We focus specifically on *lifestyles*, a concept which is familiar to medical experts (cf. widely publicized lifestyles of cardiovascular or diabetic patients) and at the same time comprehensible to the general public. We perceive lifestyles as key in the presentation of ADHD, its management and to a great degree also significant in the instigation of ADHD. We define lifestyle in terms of *distinct behavioral patterns.* Curiously, one of the best fitting definitions of lifestyle can be found in the Business Dictionary (2017): “A way of living of individuals, families (households), and societies, which they manifest in coping with their physical, psychological, social, and economic environments on a day-to-day basis.” Furthermore, we focus on comorbidities which are directly associated with respective lifestyles, i.e., obesity and food addiction, impulsive gaming, and other behaviors.

Modern science tends to consider phenomena in larger micro or macro contexts. In case of ADHD it became quite logical to focus at the microlevel of the phenomenon and a great progress is being made, e.g., in microanatomy. However, the larger social and environmental contexts of ADHD tend to be underestimated or ignored. The venture of this review is to reveal the lesser known ADHD contexts.

## Environmental, Social, and Societal Factors Relevant to ADHD

A growing body of literature indicates a relationship between ADHD and environmental, social, and societal factors. The three types of factors differ by their nature and links to ADHD but they cannot be viewed separately as they are naturally intertwined with each other. For example, all of these factors can become a source of stress and exposure to stress hormones (i.e., cortisol) and thus causally related to later ADHD (Chu et al., 2012; Samiei et al., 2015). The intertwining of micro- and macro-processes of environmental, social, and societal factors is obvious: e.g., environmental exposure to toxins is prevalent among populations with lower socioeconomic status (SES) and also among children whose mothers are less educated and less interested in healthy lifestyles. Countries which have higher rates of income inequality are also more negligent about ecological rules and environmental risks to their populations, etc. We begin by introducing the environmental factors that influence the early stages of human existence from fetal exposure to substances ingested by the mother, or environmental influences to the neonatal period.

## Environmental Factors

Exposure to environmental toxins and industrial additives have a potential negative influence on the development of the nervous system. Maleficial chemical agents and pollutants can affect the developing fetus in the maternal womb; babies are later further affected by contaminated nursing bottles, pacifiers and toys and other plastic products for daily use.

### Phthalates

Phthalates are very common as they make plastic more flexible, thus they are found in children toys, medical devices as well as in cosmetics. In animal studies exposure to these chemicals has been found to induce hormonal disturbances and developmental symptoms of hyperactivity very similar to human ADHD (Ghisari and Bonefeld-Jorgensen, 2009; Huang et al., 2009). Animal studies have found a direct correlation between prenatal exposure to industrial chemicals such as phthalates and the development of neurodevelopmental disorders as ADHD and autism (Masuo et al., 2004). A Korean study looking at the amounts of phthalate metabolites in the urine of school aged children found a significant positive correlation between higher concentrations of these chemicals and ADHD symptoms, thus confirming earlier animal studies in human samples (Kim et al., 2014). Similarly, in a study focusing on children diagnosed with ADHD, higher levels of phthalates were found in the urine of ADHD Combined and ADHD Hyperactive Impulsive children compared to normally developing children. Various neurological and behavioral differences were also noted, such as brain cortical thickness abnormalities and higher rates of externalizing disorders. Interestingly, the higher levels of phthalate metabolites were associated with the ADHD Combined and Hyperactive Impulsive children but not with the Inattentive group (Park et al., 2015).

### Bisphenol A

Similarly to phthalates, *Bisphenol A (BPA)* is ubiquitous in food packaging and other plastic products. This chemical has received media attention due to its correlation with insulin dysfunction and its mild oestrogenic effects that can influence development–especially intrauterine and fetal development. Most recently, the discovery that BPA directly influences the dopaminergic system during early development is especially relevant to ADHD and other disorders marked by impulsivity (Huang et al., 2016). As with phthalates, studies on pregnant rats exposed to BPA found increased mutations and abnormalities in their offspring; specifically, lower synaptic plasticity and disinhibition of the GABAergic system leading to a higher dopaminergic response. Symptomatically this means much higher rates of hyperactive behaviors in the rats. A particularly alarming factor of this study is that even exposure to minute doses of BPA was associated with later ADHD-like symptoms (Zhou et al., 2011). Similarly, it was found that children aged three with higher concentration of BPA in their urine displayed symptoms of hyperactivity and restlessness. The chemical was present in varying concentrations in the urine of as many as 97% of the children tested (Braun et al., 2011). A systematic review on 834 studies looking at BPA, phthalates and other endocrine disrupting chemicals (EDC) found a significant positive correlation of the exposure to these substances with both autism spectrum disorders and ADHD. It was consistently found that these chemicals cause hormonal and neurological changes that result in hyperactivity and symptoms later associated with ADHD-HI and ADHD-C (De-Cock et al., 2016). As mentioned earlier, these contaminants often play a key role in mutations related to the dopaminergic and GABAergic systems. Sagvolden et al. (2005) point to these same systems in explaining various symptoms with the dynamic developmental behavioral theory. They emphasize the special needs of these children and affirm the importance of parental training to teach them how to accomplish improvements.

There are further environmental factors involved in the development of ADHD such as nicotine, alcohol, drug, and pharmacological dependence. However, they tend to be more closely associated to social environment and behavioral lifestyles, and thus we discuss them in more details in the next segment.

## Social Factors

Many indicators suggest that ADHD is not just solely a biological phenomenon, it is also linked in numerous ways to factors of social nature. Also, exposure to many noxious stimuli is mediated socially, by friends, the lifestyles of the closest family, in particular the mother, or by their absence, i.e., by social deprivation.

*Social deprivation* or early *life stress*, which is its accompaniment, appears to be a perilous factor for the development of ADHD. Internationally adopted babies and children had higher rates of ADHD as well as lower IQ when they spent long periods of time institutionalized and separated from the biological parents. Time spent in the adoptive family had a positive effect on the IQ which had a tendency to rise to average or above average levels but curiously, the rates of ADHD persisted even after the extended length of adoption. This indicates a possible association between ADHD and the immediate environment of neonates and the effect of early life stress. However, this study did not consider prenatal exposure to drugs or other toxins from the mother (Doom et al., 2015), these will be discussed in the following paragraphs. Importantly, besides the stress of a child there is also maternal stress. Maternal stress has been found to be more associated with ADHD Hyperactive Impulsive and Combined than with the Inattentive presentation of the disorder; the study also reconfirmed the association between prenatal nicotine exposure and ADHD with comorbid Conduct Disorder (Grizenko et al., 2010).

### Maternal Lifestyles as a Gateway for ADHD Related Substances

The noxious substances which influence early child development start affecting the nervous system before the child is born. Prospective mothers may be exposed to medication, drugs, and alcohol even before they know they are pregnant, some continue in the harmful exposure also in their advanced pregnancy either because they are not fully aware of the risk involved for their child or because they are unwilling to change their lifestyles.

The toxicity of *nicotine* and *alcohol* exposure to developing fetuses is very well known. Multiple studies have linked prenatal lifestyles and addictions of pregnant mothers to the occurrence of ADHD in their children. Early nicotine exposure was found to pose a higher risk for later ADHD than alcohol and *caffeine* during pregnancy. An extensive Canadian study found that *smoking during pregnancy* was associated with ADHD and specifically a more severe presentation of ADHD with comorbid Conduct Disorder resulting in a particularly disturbed child; the study took into account both genetic and lifestyle factors and found that while the influence of genetics was significant, the lifestyle and environment had an equal influence on the development of the disorder (Sengupta et al., 2015).

The effect of alcohol yielded inconclusive and contradictory results whereas exposure to caffeine had too many other confounds (Linnet et al., 2003). In contrast to Linnet, Davis et al. (2011) found in a recent medical literature review that alcohol exposure was a major risk factor for ADHD, but even more surprisingly, they found that exposure to *junk food* including high fat and sugary food also had negative effects on the fetus. Ultimately, alcohol is transformed into sugar and therefore a diet which is high in refined sugar could also have a result similar to alcohol exposure. An American longitudinal study found that nicotine exposure in the womb was associated with ADHD Hyperactive Impulsive to a far greater degree than to the Inattentive presentation of the disorder (Gard et al., 2016). Similarly, in a Scandinavian study over 12,000 children and their mothers were observed and it was found that higher rates of obesity in the mothers were directly associated with ADHD-I in their children (Rodriguez et al., 2008). A possible explanation is presented by Davis et al. (2011) who point out similarities of high sugar consumption to alcohol intake and/or some genetic link to the disorder. Coincidentally, and as will be later described in more detail, obesity is one of the most common comorbidity of ADHD along with food addiction, with obesity starting in childhood and food addiction, respectively, at adolescence and early adulthood (Davis et al., 2011; Nigg, 2013; Cortese et al., 2016; Karaca et al., 2016).

Reviews of longitudinal data from studies on adopted babies that were brought to Scandinavia from ex-Soviet countries indicate a wide range of problems derived from prenatal alcohol exposure such as fetal alcohol syndrome and neurodevelopmental disorders. ADHD was present in approximately 51% of the children who had been exposed to alcohol prenatally (Landgren et al., 2010). Premature and very premature born babies are also at a much greater risk for ADHD, and nicotine use is very often associated with premature births (O’Shea et al., 2013).

*Acetaminophen is* one of the most common over the counter drugs administered for fever reduction and for its mild analgesic properties. A direct correlation between acetaminophen taken by mothers during their pregnancy and higher rates of hyperkinetic disorders and ADHD in their children was found in a Danish study. The researchers reviewed the records of as many as 64,322 children born between 1996 and 2002 and found consistent positive correlations with acetaminophen exposure and ADHD. Acetaminophen was unsurprisingly also found to have endocrine disrupting properties (Liew et al., 2014). One of the most recent studies on the topic found that prenatal exposure to acetaminophen is associated with adverse effects on neurodevelopment and raised the risk of autism as well as ADHD. This study was based on parental interviews and frequency of administration of the drug by the mother, a positive correlation between exposure and various adverse effects was found (Avella-Garcia et al., 2016). Others have criticized the designs of similar studies, arguing there is too much space for confounding factors as they are based on parents’ self-reports, yet they acknowledge animal studies that have shown neurological effects in regards to acetaminophen exposure but argue that these mutations may not apply to human subjects (Fays et al., 2015).

### Parenting Styles and ADHD

The way parents raise their offspring is crucial to understanding most aspects of mental health as well as later lifestyles. It can be argued that each type of comorbidity and lifestyle can be at least to some degree influenced by parenting. From very early on, individuals who later develop ADHD show an irritable temperament. Ineffective, inconsistent, and especially negligent parenting were found to exacerbate ADHD symptoms and be predictive of later disruptive behavior disorders such as Conduct Disorder; furthermore, children who had more positive and involved parenting manifested an improvement of symptoms (Ullsperger et al., 2016). ADHD in its childhood presentations is not only directly influenced by parenting styles but also by parental perception.

Generally speaking, there are predictive and protective factors linking substance abuse and parenting. One of the most widely recommended parenting style with respect to ADHD is authoritative parenting. This parenting style includes warmth, communication, and clear boundaries for the child. This style of parenting is compared with authoritarian which is less warm, with strict boundaries, low communication, and strict rules. Permissive parenting consists of low boundaries and varying levels of communication where the parental role is not clearly defined; finally, there is negligent parenting (laissez-faire), where the child is not being properly taken care of and the parents are often missing from the households (Briesmeister and Schaefer, 2007; Schaefer, 2007). A systematic review of parenting styles and drug abuse consistently found a clear correlation with authoritative parenting and lower rates of drug abuse and addiction (Becoña et al., 2012). In a South African study, authoritative mothers in families with children with ADHD had much better communicating styles and better educational outcomes for their children (Tancred and Greeff, 2015). Similarly, severity of symptoms as well as academic and social well-being of children aged 7–11 years with ADHD Inattentive presentation, were directly correlated with parenting: negative parenting such as harshness and/or neglect was associated with worse outcomes (Haack et al., 2016).

One of the most classically acclaimed examples is the Baumrind (2013) study that found that children raised with an authoritative parenting style in the United States not only had lower drug and alcohol abuse incidences but were more competitive and successful in their academic careers. In the United States, authoritative parenting is consistently advised and considered ideal for healthy outcomes in psychological development as well as a good preventive measure for substance abuse and addiction. Interestingly, European scholars favor a contrasting attitude: indulgent (permissive) parenting turned out equally effective.

Strict authoritarian parenting as well as negligent parenting were the most widely implicated parenting styles associated with drug and alcohol abuse (Calafat et al., 2014). A Canadian study focusing on the families of children with ADHD found that not only were ADHD cases most likely among mothers with children with ADHD but that a major component was family dysfunction. Hostility and parental conflicts were correlated with ADHD as well as single parent households (Williamson and Johnston, 2016). However, correlational studies usually cannot exclude a reverse causality, i.e., the disruptive child might have severed the bonds of a stressed family.

Studies looking into the parenting behaviors of adults with ADHD found significant differences in self-reports and observations about their own relationship and parental style with their children. ADHD Inattentive parents were most likely to self-report negative parenting, as they were consistently ignoring their children and getting easily annoyed with them. In the Hyperactive Impulsive group much more positive interactions were observed, in the self-report ADHD parents had a tendency to conflate their positive parenting to higher self-esteem or a result of exaggeration due to other comorbid mental illnesses often seen in ADHD-HI like Antisocial Personality Disorder (Lui et al., 2013).

The family is the most influential environment where children’s behavior is influenced and habits are formed (Vandewater and Lansford, 1998; Day, 2010; Ptacek et al., 2014a). A functional and supportive family can help to prevent ADHD onset and in case ADHD cannot be prevented, family members may mitigate the severity of symptoms.

## Societal Factors – Socioeconomic, Cultural and Political Aspects

While suffering from ADHD appears as a highly individualized private matter, the intervening factors may be of a general societal nature. Poverty and lack of access to resources are at the forefront of societal phenomena quoted in the literature.

Lower SES and trends toward non-traditional single parent households (Klein et al., 2012; Russell et al., 2016) have been linked to an increased ADHD incidence. Lower SES has also been associated with other ADHD-prone conditions, such as higher risk of disease and higher exposure to pollution (Woolf et al., 2015), higher smoking and drinking rates (Bader et al., 2011; Woolf et al., 2015), i.e., lower SES is generally linked to unfavorable prenatal conditions, unhealthy lifestyles and nuclear family problems discussed in the previous section on social factors. Individuals with lower SES have less access to resources, including proper food, lack of economic means to pay for decent education. Poverty is stressful and stress, as mentioned in the beginning of this section, is a general ADHD risk factor. While particularly noxious during the early stages of development, stress has been associated consistently with a later onset of ADHD as well as comorbid asthma (Grizenko et al., 2015). Lower SES is often associated with poor family cohesiveness and frequent family conflicts (Conger et al., 2010; Santiago et al., 2011). This creates an unhealthy environment for children to grow up in and puts them at risk of a variety of problems including ADHD. A study comparing 100 children aged 6–18 years who had ADHD Inattentive presentation (ADHD-I) to a control group of 100 children indicated that family problems and conflicts are one of the most common risk factors for ADHD-I. The researchers used both the Family Adversity Scale (FAS) and the Family Environment Scale (FES) to determine family cohesiveness and problematic interpersonal relationships in the household. Children who scored high on the FAS and low on the FES were consistently from the ADHD-I group, showing that family adversity situations and conflicts are a risk factor. These are associated with low SES and lack of resources which the study showed are predictive factors, too (Pheula et al., 2011). A meta-analysis found a significant correlation between ADHD and lower SES. When compared to children from middle income families, those from poor families were 1.85–2.3 times more likely to suffer from ADHD.

Still, the issue of poverty, status and ADHD is not without controversy. While ADHD does typically correlate with low SES, some perceive an opposite tendency: ADHD appears to be almost a fashionable, trendy label in advanced societies. It is most prevalent in the USA and Western Europe, much less so in developing countries. The rest of the world is trying to catch up with the United States; e.g., a noted sociologist, P. Conrad–was quoted by the Huffington Post article by Gregoire (2014): “With millions more kids (and adults) likely to be diagnosed with and treated for ADHD in the next decades we see the export of American behavioral norms worldwide. This may be more insidious than the globalization of American fast food or pop music, in that it comes in the name of proper mental health and behavior.” The Huffington Post stressed that the ADHD disorder “has become a cultural and economic phenomenon–but it may not be a medical one....” with a significant presence of the pharmaceutical companies and their lobbyists. We are facing a moral dilemma: a growing conflict between the humanistic effort to help to diagnose and manage ADHD problems and medicalization of the disorder around the world (Conrad and Bergey, 2014).

## Environmental, Social and Societal Factors in Interaction

The question still remains to what degree the poor economic status is the result of societal factors (the economic system, structural racism, structural violence, lack of resources, etc.) or the result of parents’ mental health, genetic factors and others. A consensus tends to be in a conclusion that multiple factors are important at the same time (Russell et al., 2016). Extensive reviews confirm this assumption by finding consistent correlations between socioeconomic factors and ADHD both in children and their families (Bernfort et al., 2008).

Lifestyles related to ADHD are easily acquired within the family as social learning would entail. A longitudinal study on ADHD found low SES to be one of the greatest risk factors for ADHD. Furthermore, this was associated with parental behaviors that are easily learned such as higher rates of smoking and eating junk food as a normal staple food (Russell et al., 2016). In a cross national review focusing on a smoking cessation program in Europe, it was found that higher rates of smoking were much more frequent in individuals from a lower socioeconomic background (Bosdriesz et al., 2016). It is therefore clear that the associations between class and social factors have a strong implication in ADHD development and its associated lifestyles. Higher rates of smoking, drug abuse, and poor diet are all included as lifestyles and at times comorbidities associated with ADHD (Nigg, 2013).

In summary, it is important to keep a holistic point of view and not to underestimate the extrinsic factors influencing this disorder such as SES and the effect of certain lifestyles mothers had before the individual was born.

The above mentioned effects are all significant and at times preventable but it is important to keep in mind that these lifestyles and higher exposure to toxic chemicals and pollution are also generally associated with lower SES and the social problems that are associated with poverty (Duncan and Brooks-Gunn, 2000; Woolf et al., 2015) which will be discussed later in more detail.

## Lifestyles and Comorbidities Associated with ADHD

Attention Deficit Hyperactivity Disorder is associated with various peculiarities in lifestyle and resulting comorbidities which vary with the different manifestations of the disorder and present themselves in various developmental stages of life. The comorbidities are both somatic and/or behavioral, we will focus primarily on those that are related to certain behavioral manifestations associated with the disorder such as addictive gaming and impulsive eating leading to obesity and addiction.

From the neonatal stage there is an association between certain particularly irritable temperaments and later negative emotive expression in pre-school. Many of these children are later diagnosed with ADHD (Rabinovitz et al., 2016).

## Comorbidities in Various Presentations of ADHD

Attention Deficit Hyperactivity Disorder is not a uniform disorder, it consists of three clinical presentations; Hyperactive Impulsive (HI), Inattentive (I), and Combined Hyperactive Inattentive (CHI). The various presentations of ADHD differ in comorbidities (Wilens et al., 2009; Grizenko et al., 2010). This is particularly confounding and problematic as clinicians don’t often properly differentiate among the various types of ADHD. The genetic and behavioral differences between Inattentive, Hyperactive and Combined ADHD patients were so vast to lead some researchers to see them as separate diagnoses with differences even in reactions to methylphenidate which is universally prescribed for all presentations of the disorder (Grizenko et al., 2010, 2015). Others have differentiated the dopaminergic functions and GABAergic effects on the different symptoms such as impulsivity and hyperactivity (Sagvolden et al., 2005). In the Hyperactive Impulsive presentation of ADHD, comorbidities such as conduct disorder in childhood and antisocial personality traits in late adolescence were more common.

The Inattentive presentation of the disorder showed different comorbidities like depression and anxiety. Furthermore, the individuals showing higher inattentive presentation of the disorder manifested more frequent social isolation and lack of intimate relationships (Halfon et al., 2013; Barkley, 2016). Obesity, another major comorbidity was prevalent among the inattentive group but not among the Hyperactive Impulsive group (Halfon et al., 2013).

Nicotinism, alcohol abuse, and drug abuse were all noted pathological lifestyles and comorbidities in the ADHD Hyperactive Impulsive “subtype” as it was defined in the Diagnostic and Statistical Manual of Mental Disorders IV-TR. Nigg (2013) among others points out that the comorbidities of ADHD are extremely important for the clinician to know as they constitute most of the health issues and dangers associated with the disorder. Gillberg et al. (2004) urge that clinicians working with ADHD should have ample knowledge of neurology as well as the vast arrays of comorbidities associated with the disorder, as it is almost impossible to work with the disorder as a single cause as the lifestyles are often the main symptoms.

### Conduct and Personality Disorders

Perhaps the most serious aspect of ADHD lies in its tendency to be associated with disorders, some of which impact not just behavior but personality. This endangers not just the well-being and life of the ADHD sufferers but also of their social environment. These comorbidities are seen in children and if not resolved by late adolescence can then become personality disorders such as antisocial PD or continue as externalizing disorders into adulthood.

In a sample of 358 Australian children diagnosed with ADHD, 92% had other comorbid health conditions along with parents suffering from some disorder (Silva et al., 2015). As was previously mentioned, in the earliest stages of human existence, maternal stress (and resulting premature birth) and/or exposure to nicotine are factors that have been consistently associated with ADHD (Chu et al., 2012; Nosarti et al., 2012; Grizenko et al., 2015; Samiei et al., 2015) along with other mental disorders (Glaslova et al., 2004). The study by Samiei et al. (2015) closely analyzed the social aspects (see also SES factors above) of the disorder and left the question open about whether or not it was the stress of the mother that caused ADHD or whether a vicious cycle has developed, in which the child’s disorder and behavioral symptoms were responsible for the increased stress of the parents. In practical terms, the authors argue that psychological care and adequate doctor-to-patient communication is equally important for the parents of children affected with the disorder in addition to the children’s treatment (Samiei et al., 2015).

Attention Deficit Hyperactivity Disorder is commonly associated with other externalizing disorders (Gillberg et al., 2004; Silva et al., 2015). These are mental disorders that have clear and often problematic behavioral implications due to socially unacceptable and/or antisocial behavior that constitutes them. These disorders include ODD and Conduct Disorder in children. They also include personality disorders in adults including Antisocial Personality Disorder and Borderline Personality Disorder, as well as substance abuse disorders and other disorders or behaviors such as kleptomania that can lead to criminal charges. Externalizing disorders are currently not listed in the DSM-5 but were proposed as the description is very useful to identify certain disorders with problematic behavioral traits (Krueger et al., 2005).

### Sleep Disorders

Sleep disorders are another comorbidity that affect children with ADHD at a much higher level than normally developing children. A meta-analysis review evaluating studies from 1987 to 2008 found consistent differences such as bedtime resistance (defiance to parents), sleep onset difficulties, night awakenings and at times low sleep duration (Cortese et al., 2009). Other studies have pointed out some subtle differences in relation to the circadian rhythm and melatonin in children with ADHD, thus explaining the often-noted sleep onset difficulties (Nováková et al., 2011). Furthermore, these sleep disturbances can further aggravate the symptoms of ADHD such as inattention and motor skill dysfunction (Schneider et al., 2016). Kirov and Brand (2014) argue that the influence of sleep in the symptomatology of ADHD is one that needs much further research and is an area often neglected. Owens et al. (2013) similarly report on a multidisciplinary approach of various physicians to investigating the many aspects of sleep and ADHD to develop new therapies related to sleep in order to manage ADHD indirectly. Indeed, in an Iranian study on sleep interventions in children with ADHD it was found that the children whose parents were trained to implement better sleeping routines for their children had significant improvement in their physical, emotional, and mental well-being; and interestingly had improved interpersonal relationships among peers (Keshavarzi et al., 2014).

### Obesity

Obesity has been linked with ADHD both in childhood and adulthood in several major longitudinal studies and meta analyses, making it one of the most common comorbidities of ADHD with males being more afflicted with the condition (Cortese et al., 2013; Ptacek et al., 2014b; Cortese et al., 2016). Incidentally, the DRD 4 genetic pathway, is correlated both with ADHD (Ptacek et al., 2011a; Choudhry et al., 2013) and other impulsive disorders like alcoholism and drug addiction, a genetic link and shared genes for ADHD and obesity have also been isolated. ADHD is often associated with motor skill dysfunction from an early age. In a cross-sectional study, it was found that motor dysfunction was very often associated with childhood obesity, but children with just ADHD were most likely to be underweight, which was paradoxical for the researchers. This has led to the conclusion that when studying this condition, it is extremely important to differentiate and assess the conditions separately (Goulardins et al., 2016).

A longitudinal study found very strong links between childhood ADHD with both Hyperactive Impulsive and Inattentive showing greater risk for being overweight or obese, respectively. The study found a stronger correlation in the Hyperactive Impulsive group with later hypertension than with obesity, possibly due to lifestyles associated with this presentation such as higher consumption of nicotine and other drugs (Fuemmeler et al., 2011). The extremely high rate of comorbid ADHD among the obese was noted in a study in Israel where the researchers strongly advise physicians to check for or refer obese patients to clinical psychologists to assess ADHD and other mental health disorders, as curing lifestyles may improve both conditions (Agranat-Megedger et al., 2005). In contrast, a large cross-sectional study with a sample of 45,987 individuals aged 10–17 years in the United States found that the individuals with comorbid ADHD and a learning disorder, along with the youth with learning disorder alone were more likely to be obese than their peers (OR = 1.464 and OR = 2.094, *p* = 0.01). However, the study did not find an association between children with only ADHD and obesity (OR = 0.870). Those with ADHD and not obesity were found to have lower levels of physical activity, furthermore, the study did not differentiate among the clinical presentation of the disorder (i.e., inattentive, combined, etc.). After accounting for demographic factors (e.g., gender, age, ethnicity, and SES), ADHD patients with comorbid learning disability that were medicated had lower obesity rates than non-medicated patients (OR = 1.393 and OR = 2.516, respectively). This study suggests that ADHD symptoms alone are not associated with obesity, and points toward a different possibility: the presence of other comorbid psychiatric disorders and or social factors. The study supports the lower incidence of obesity in medicated patients, but the length of time of drug treatment or the type of drug used (i.e., methylphenidate or a non-stimulant) was not reported (Cook et al., 2015). We hypothesize that in many of the obesity and ADHD correlation studies, medication could be a confounding factor in getting correct results and correlations. This is due to the fact that most drugs used to manage ADHD are of the stimulant class such as methylphenidate and amphetamine, both have the effect of speeding up the metabolic rate. Reviewing the literature, we found a great amount of studies failed to register the duration and at times whether drugs were being administered while analyzing the correlations between obesity and ADHD. At other times, comorbidities in childhood such as ODD or Conduct Disorder were not taken into account. The long term metabolic effects of ADHD drugs need to be studied to a greater extent in relationship to metabolic rate and long term changes, rebound effects and tolerance (Weissenberger and Akotia, 2015). In fact, some researchers are even calling for stimulants such as methylphenidate and amphetamine to be used both for the management of ADHD symptoms and subsequent obesity and food addiction improvements (Poulton et al., 2016). It is crucial to note that some studies have also mentioned a much higher prevalence of obesity among the predominantly inattentive presentation of ADHD but not so much in the Hyperactive Impulsive group (Fuemmeler et al., 2011; Khalife et al., 2014).

A clinical study in Turkey found that children and teenagers with ADHD have a much lower tolerance to stress and frustration than normally developing children and thus may binge on junk food possibly as a form of self-medicating. In addition to this, ADHD was correlated with both malnutrition as well as being overweight and obese. The researchers state that this shows ADHD to be a major risk factor not only for obesity but also for eating disorders in general (Güngör et al., 2016). The lower threshold for stress as well as emotional dysregulation and behaviors associated with this have also been noted in a meta-analysis by Barkley (2016). A Korean study further linked bulimia, higher body mass index and obesity with ADHD as well as lower SES (Kim et al., 2014). ADHD has been disparagingly studied among males but among females it is often associated with eating disorders like bulimia nervosa and binge eating (Ptacek et al., 2016). There is also a separate question of the possible effect of ADHD on physical growth and development (Ptacek et al., 2009a,b, 2014b) and may be contributing to above mentioned questions.

### Food Addiction

Just as in drug addiction or gambling, an unhealthy preoccupation with food along with compulsivity to eat is often associated with ADHD. This begins in childhood and continues into adulthood if the disorder persists often leading to comorbid obesity (Cortese et al., 2013). Generally, individuals and animals will binge on high fat and salty food or high fat and sugary food, this has a dopaminergic effect that is similar to a drug of abuse (i.e., nicotine, cocaine). Food addiction is now a recognized mental health condition and there are standardized scales to measure it such as the Yale Food Addiction Scale. Davis (2011) pointed out that in an obese population the food addicts were most likely to have comorbid ADHD and those found with these behaviors had higher emotional instability and were displaying the classic behavior noted in other addictions such as using the food to soothe unpleasant states of mind as seen in traditional drug users; the food was also being used to self*-*medicate certain unpleasant states of consciousness such as anxiety and sadness. The study confirmed a new type of obese patient or subtype that was not previously officially recognized by the medical field aside from anecdotal accounts, the food addict. It must be noted though, that due to the high-risk factor associated, those with ADHD both in childhood and adulthood are most likely to abuse drugs, food, and electronic media (Nigg, 2013; Karaca et al., 2016; Yen et al., 2016). A Canadian research team pointed out in detail how ADHD may not only be one of the most important risk factors for developing obesity but also one of the driving forces behind the impulsive eating previously mentioned. In concord with Davis et al. (2011), they argue that the treatment and improvement of ADHD symptoms will directly reduce obesity and result in weight loss. They recommend tools such as the Yale Food Addiction Scale along with the classical self-assessment scales administered for ADHD as the two are often intrinsically connected. Behavioral modifications are recommended in treating ADHD and its subsequent comorbidities such as food addiction (Yang, 2010). Pharmacological interventions have also been successful in managing hedonic and impulsive eating habits in those affected with the disorder, ideally together with psychotherapy (Hanan, 2016). Throughout the literature, there is a pervasive common emphasis on immediate gratification, poor discipline and self-control with the individuals suffering from ADHD. Addictive behaviors are among the most prominent behavioral tendencies associated with the disorder which can lead to pathological comorbidities such as various types of addictions and dependencies (Karaca et al., 2016).

Parenting is also directly involved in comorbid obesity of ADHD children. It was found that harsh parenting in adolescence or, more specifically, an authoritarian style as well as food insecurity due to lower SES (again connected to ADHD) correlate strongly with later obesity (Lohman et al., 2016). A longitudinal study that took place in Canada from 1994 to 2008 and focused on parenting similarly found that authoritarian and negligent parenting were directly associated with obesity. When compared to the children raised in authoritative and traditional homes, the children from authoritarian homes were 35% more likely to be obese and the children from negligent homes 44% more. The authors clarify that the correlation is certainly there but that the effects of poverty (SES) should not be underestimated, specifically when looking at children from negligent homes (Kakinami et al., 2015). In contrast, behavioral modification therapy for obese children found that warm and authoritative parenting had a very positive effect in reducing the body mass index and raising the quality of life of obese children in recovery (Rhee et al., 2016). The entire body of studies show the great importance of family cohesiveness in this disorder and point to often ignored extrinsic social factors, that we argue are equally if not more important than the genetic factors involved.

### Substance Abuse

Attention Deficit Hyperactivity Disorder is often associated with substance abuse and addiction. It has been estimated that almost one quarter of those suffering from Substance Use Disorder (SUD) have comorbid ADHD and they furthermore have a much worse prognosis in treatment compared to non-ADHD substance abusers (Van Emmerik-van Oortmerssen et al., 2015). Differentiating the presentation of the disorder is crucial; in a study on cocaine addicted and nicotine addicted adults with ADHD compared to other non-addicted individuals diagnosed with ADHD, both the cocaine and nicotine addicted patients scored very high in the hyperactive/impulsive presentation scale and very low in the inattentive both in adulthood and in a self-report childhood scale (Saules et al., 2003). Similarly, in one of the best known longitudinal study on ADHD conducted in a 33-year period, higher rates of nicotine addiction and substance abuse were noted in the adults who showed what we now classify as Combined Presentation of the disorder in childhood (Klein et al., 2012). Childhood ADHD was found to be associated with higher levels of later sensation seeking.

A Swiss study found that young adults (mean age of 20) with ADHD and comorbid Antisocial Personality Disorder had been experimenting with legal and illegal drugs from an early age. The study found higher rates of cocaine and nicotine use/addiction, but not as much alcoholism compared to controls (Estévez et al., 2016).

In a Danish longitudinal study looking at gender and its influence on SUD found that unlike in the general population, the rate of individuals with SUD and ADHD was at times higher in females. Different comorbidities were also reported in the female population such as higher rates of depression, pointing out that although ADHD is more common in males than in females, the disorder is just as disruptive in females although it may have a different and more masked presentation (Ottosen et al., 2016). In contrast to this study, more anomalous findings were observed; specifically Davis et al. (2015) found much higher rates of drug and alcohol abuse among those with ADHD compared to the general population but no gender differences in contrast with what is seen in non-ADHD populations.

Substance abuse and alcoholism are widely known to be running in families, and research shows both environmental and genetic causes (Thapar, 2015; Kendler et al., 2016). A meta-analysis in Sweden reviewing substance abuse and addiction among ADHD populations and their relatives, found a strong correlation among the two. The researchers argue that this is due to genetics, as the genetic pathways responsible for ADHD are the same as those involved with proneness to addiction (Skoglund et al., 2015). The dopamine receptor gene DRD2 that is associated with ADHD Hyperactive Impulsive presentation was also found to be associated with alcoholism and drug addiction (Jones et al., 2015). A study at a rehabilitation facility for problematic gamblers found that one third of the inpatients came out positive during assessment for ADHD and the majority of those with ADHD had Cluster B personality disorders as a comorbidity with no major differences among sexes (Waluk et al., 2016). Another factor related to drug abuse and other addictions is time perspective. It has been hypothesized that individuals with ADHD Hyperactive Impulsive have a present hedonistic time orientation as defined by Philip Zimbardo (Weissenberger et al., 2016).

### New Addictions – Electronic Media Dependencies

Similarly to drug addiction, currently we see more cases of compulsive internet use, social media addiction and video game addiction; we refer to these as the new addictions. A French study tested almost 1000 college students with five assessment inventories including the Wender-Utah, ASRS-V1, UPPS, Rosenberg Scale and CPPG to assess both ADHD in childhood and in adulthood. It revealed that the Adult ADHD Hyperactive Impulsive presentation is highly correlated with problematic gambling and had the highest rates of video game addiction (Romo et al., 2015).

As with the other lifestyles and comorbidities, there are gender differences in problematic computer use and psychiatric disorders. In some cases, even paradoxical ones, for instance a study in Taiwan looking at which type of ADHD presentation and gender is more likely to show higher rates of internet addiction, found that those with higher inattentive symptoms and females with ADHD were most likely to be addicted to the internet (Yen et al., 2009). Subsequent analyses showed that females suffering from ADHD and/or other disorders such as Borderline Personality Disorders were most likely to be addicted to social media use. Males with higher impulsivity symptoms on the other hand were found more likely to fall for the addictive game usage. The male group who came out as highest on the ASRS scale for ADHD had one of the strongest correlation for impulsive gaming. The gender difference was emphasized by the researchers to also point out the fallacy of the previously proposed Problematic Internet Use (PIU) diagnosis as too broad and encompassing as the behaviors are varied with this type of addiction (Andreassen et al., 2016). The PIU description/diagnosis was proposed as an affective disorder in the early 2000s to be added to the DSM consisting of an unhealthy preoccupation with the Internet as well as impulsive and addictive use of the internet not specifying exactly for which purposes (i.e., social media or gambling) but eventually was not added (Shapira et al., 2000). Regardless of the nomenclature, the link with unhealthy technological habits in people with ADHD is prevalent. ADHD was one of the most common risk factors for impulsive internet use and Internet Gaming Disorder (IGD), with neurochemical alterations noted in one ADHD study comparing compulsive gamers with diagnosed ADHD and without the diagnosis (Hyun et al., 2015; Karaca et al., 2016; Bae et al., 2016).

Similarly, Yen et al. (2016) found a significant correlation between impulsive gaming disorder and ADHD, the researchers also assessed impulsivity and aggressiveness and found a strong correlation with it in individuals diagnosed with ADHD but not in those who were only impulsive gamers. High impulsivity was most common among males as was previously mentioned. In terms of time perspective, the majority of those with a Facebook addiction tended to score high in Past Negative and Present Fatalistism in the Zimbardo Time Perspective Inventory. Present hedonism was associated with addictive video game use and pornography usage, but not necessarily social media or Facebook addiction. Gender was also a factor, with females being more attracted to social media use (Przepiorka and Blachnio, 2016).

## Discussion and Conclusion

Attention Deficit Hyperactivity Disorder is a common neurodevelopmental disorder. Our article is devoted to a variety of factors which are involved in its onset and presentations; we paid particular attention to factors of an environmental nature spanning from chemical to societal agents which are often neglected – see (**Table 1**).

**Table 1:** Examples of the environmental agents (chemical, social, and societal) relevant to the onset of Attention Deficit Hyperactivity Disorder (ADHD).

	Prenatal stage (maternal exposure)	Nursing stage	Childhood	Adolescence	Adulthood
Chemical agents	Nicotinism and other substance abuse; exposure to EDC (endocrine disrupting chemicals), acetaminophen, unhealthy diet	Second hand smoke; EDC contaminated bottles and toys	Second hand smoke; EDC contaminated toys; unhealthy diet	Substance abuse; unhealthy diet	Substance abuse; unhealthy diet
Social agents	Maternal stress	Early life stress, social deprivation; inconsistent care	Family dysfunction, harmful parenting styles (authoritarianism, negligence), negative role models	Negative peer influence and authorities condoning harmful addictive lifestyles	Social mediation of noxious stimuli and harmful lifestyles
	Extreme stress (stress hormone exposure)				
Societal agents	Poverty – higher exposure to toxins, inadequate healthcare and education, higher stress from family and immediate environment				

Accompanied by behavioral manifestations, lifestyles and comorbidities make the life of the patients and their families difficult. The lifestyles appear both in the role of precursors and symptoms – see (**Table 2**).

**Table 2:** The dual role of harmful lifestyles, precursors and symptoms.

Precursors: Lifestyles *contributing* to ADHD	Lifestyles related to ADHD *presentation*
Unhealthy maternal lifestyles causing exposure to noxious agents and stress; harmful parenting styles; parents and peers involved in harmful lifestyles (addictive behaviors, models of lack of self-control).	Lack of self-control, tendency to succumb to addictions; substance addictions; food addictions (addictions to high volume and low quality food – mainly easily accessible fast food); social isolation and dependency on electronic media, impulsive gaming (prevalently in males); oppositional behavior and aggressiveness; passive lifestyle (mostly in inattentive ADHD); depressed passive lifestyle (often in females); vicarious social life on social media networks (often in females)

Lifestyles are often perpetuated in families and sometimes directly cause vicious cycles; e.g., mothers are exposing their unborn children to nicotine and raising the risk of the disorder in their child. We emphasize the significance of lifestyles associated with the disorder and we suggest for clinicians to treat these with equal attention as the disorder itself. With the fairly recent advent of Adult ADHD as a new recognized diagnosis in the DSM-V, some researchers are going so far as to say that childhood and adult ADHD are at times not related (Faraone and Biederman, 2016). This view may seem very controversial, but an emphasis on the lifestyles of these adults could be crucial to be understanding ADHD that starts in adulthood. Much more research will be needed on this topic as the disorder was long considered a “childhood disorder.” As pointed out in the article, the disorder only carries out into adulthood approximately 50% of the times (Moreno-Alcázar et al., 2016). That view is not consistent with a notion which classifies the disorder as “lifelong.” Due to its behavioral implications with lifestyles burdened by substance abuse and impulsive game disorder, the possibility that the lifestyles can be a later factor in developing the disorder is a controversial topic that we suggest more research be devoted to in the psychiatric field.

Our analyses lead us to a growing conviction that with respect to all ages of ADHD patients, both diagnostics and treatment should encompass a wide, holistic approach. This approach should not neglect social and economic factors. Low SES and high stress (and related exposure to noxious substances and lifestyles) have been consistently shown to greatly contribute to the disorder from the prenatal period on. Adequate communication by clinicians and an awareness of these factors is very important. Pediatricians and General Practitioners should be well aware of the environmental risks to the developing fetus and warn the prospective mothers about preventable hazards.

We strongly recommend that new research includes healthy lifestyle, enhancement of family communication, as well as sound societal policies besides inevitable focus on the neurological substance of the disorder. One positive change for educators and schools is to have longer outdoor time for children and emphasize the importance of physical education as this has been very helpful for hyperactive and ADHD children (Pontifex et al., 2013). Additional research on the environmental pollutants and their effect on the disorder and publication of the findings may in the long run also pressure citizens to demand companies to pursue more responsible practices, as was seen in the public backlash against products with BPA. Open minded physicians and clinicians with a holistic perspective will better understand and more effectively improve the quality of life of their patients and of those around them.

## Author Contributions

SW: Contributed to ideating the review, designed and literature review. RP: Supervised the work contributed to literature review. MK-B: Contributed to literature review. AE and KS: Contributed to literature review and formatting. JR and MG: Contributed to supervision and literature review.

## Conflict of Interest Statement

The authors declare that the research was conducted in the absence of any commercial or financial relationships that could be construed as a potential conflict of interest.
